# Wernicke’s Encephalopathy Associated With Transient Gestational Hyperthyroidism and Hyperemesis Gravidarum

**DOI:** 10.7759/cureus.10012

**Published:** 2020-08-25

**Authors:** Shakeel Ahmed, Dania Ahmed, Salem Abo Salah, Joe Mathew, Zohaib Yousaf

**Affiliations:** 1 Pulmonology, Hamad Medical Corporation, Doha, QAT; 2 Medicine, College of Physicians and Surgeons Pakistan, Karachi, PAK; 3 Medicine, Hamad Medical Corporation, Doha, QAT; 4 Internal Medicine, Hamad Medical Corporation, Doha, QAT; 5 Clinical Research, Dresden International University, Dresden, DEU

**Keywords:** wernicke's encephalopathy, pregnancy, hyperemesis gravidarum, hyperthyroidism, transient gestational hyperthyroidism

## Abstract

Wernicke's encephalopathy (WE) is a potentially reversible yet severe neurological manifestation caused by thiamine (vitamin B1) deficiency. It is commonly associated with heavy alcohol consumption. Other rare causes include severe and prolonged vomiting, starvation, and prolonged intravenous feeding. WE patients usually present with the triad of ocular signs, ataxia, and confusion. In non-alcoholic patients, the full classic triad develops in 10-16% of cases. Characteristic MRI findings and clinical response to thiamine confirm the diagnosis. In this report, we present a case of WE in the setting of transient gestational hyperthyroidism and hyperemesis gravidarum (HG).

## Introduction

Wernicke's encephalopathy (WE) is a serious neurological disorder secondary to severe thiamine deficiency [1]. It is mostly prevalent among alcoholics (12.5%), and is difficult to recognize in non-alcohol-related conditions, including prolonged starvation and post-bariatric surgery. It is rare in severe hyperemesis gravidarum (HG), with a prevalence that ranges from 0.04 to 0.13% [2].

WE occurring due to hyperthyroidism is very rare. The prevalence of hyperthyroidism secondary to primary thyroid disease is low in pregnancy. However, transient gestational hyperthyroidism is associated with HG and observed in about 60% of patients [3,4]. A high level of circulating human chorionic gonadotropin (hCG) causes clinical thyrotoxicosis [5,6]. We present a case of severe WE in a female non-alcoholic inpatient that was precipitated by inadequate nutritional intake due to HG and development of thyrotoxicosis.

## Case presentation

A 32-year-old female, who was 12 weeks pregnant, presented with a history of confusion and abnormal behavior for one day. She had experienced recurrent vomiting for the past one month. She had been on a liquid diet for the past month due to severe vomiting. She had received intravenous dextrose infusion from her primary care physician. She had no history of fever, drug ingestion, or acute illness. Her past medical history was negative for any chronic medical conditions.

On clinical examination, she appeared unwell and dehydrated. She was afebrile, having sinus tachycardia (112/min) with a blood pressure of 117/65 mmHg. She was confused and disoriented. She had ophthalmoplegia, hyporeflexia, and ataxic gait. There was no neck stiffness. The rest of the systematic examination was unremarkable.

**Figure 1:**
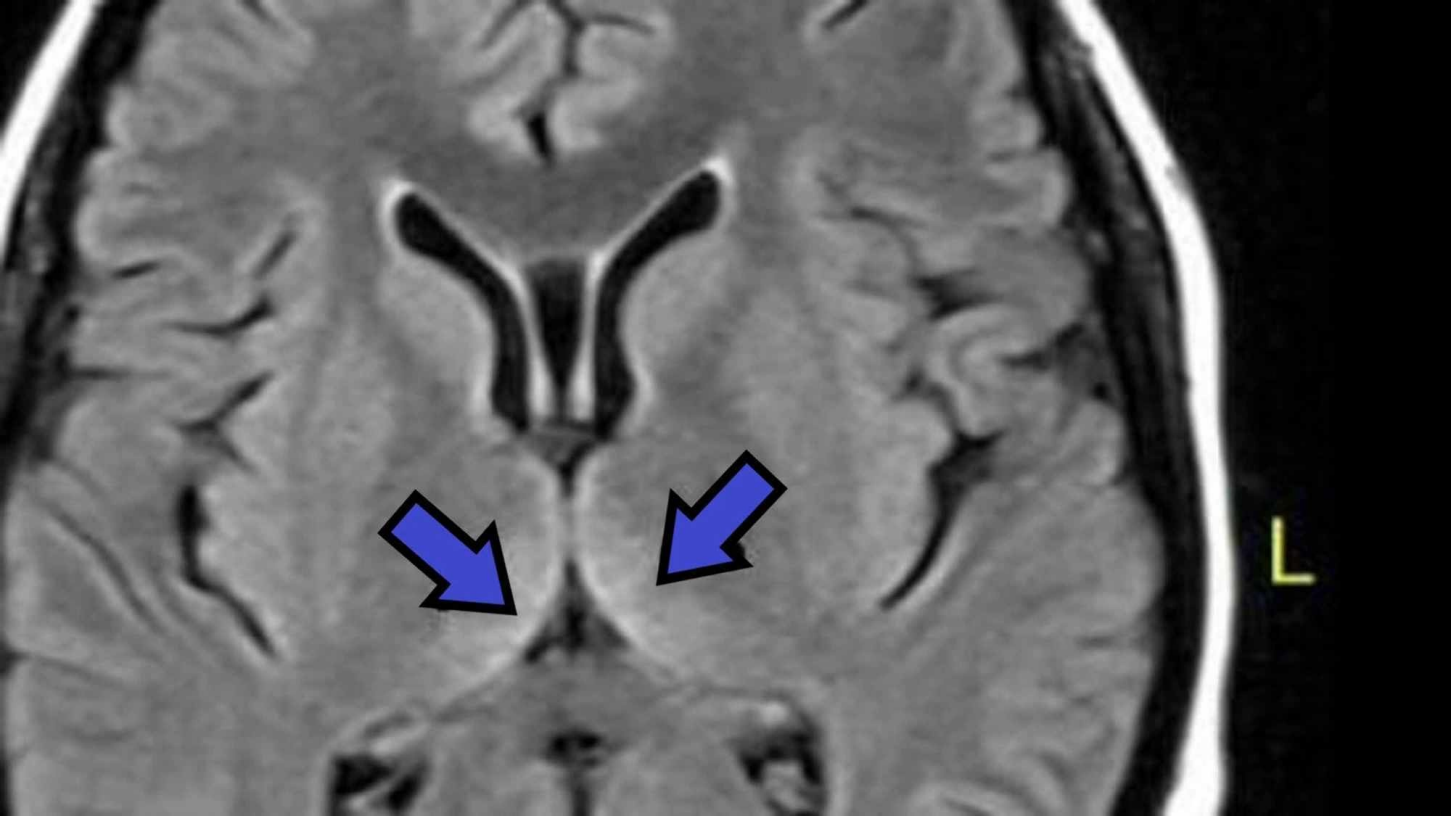
Hyper-intense signals in the medial thalamus (arrows)

**Figure 2:**
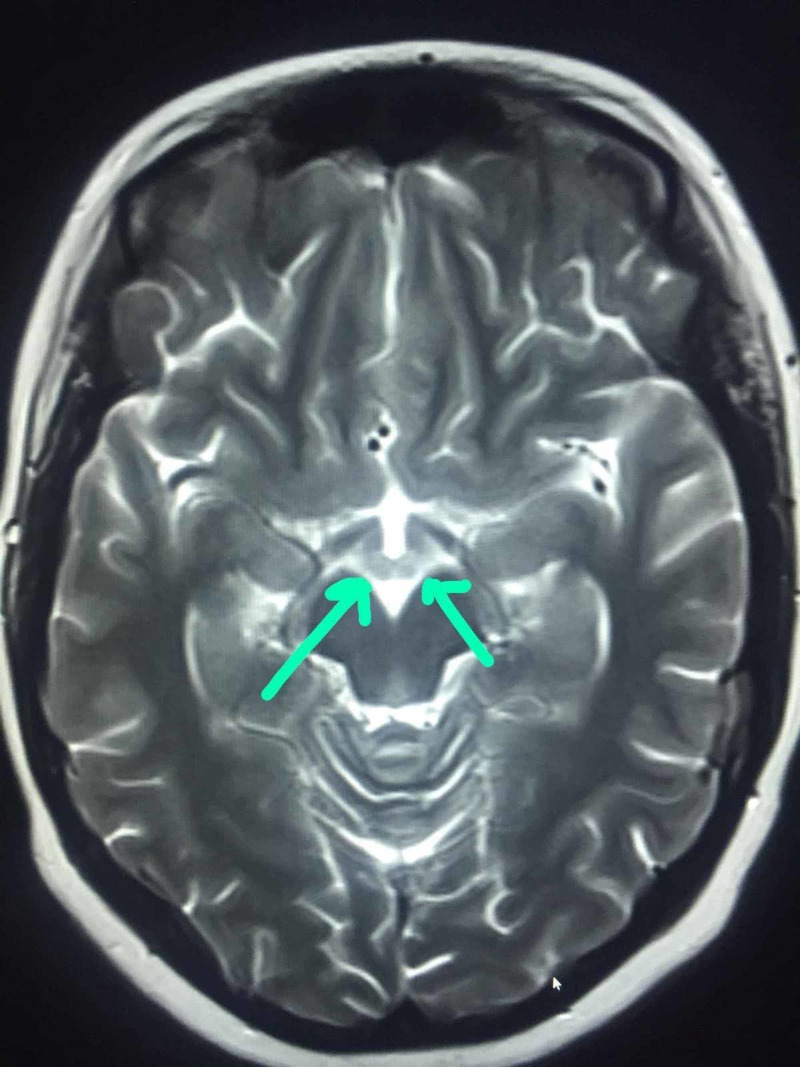
Hyper-intense signals in mammillary bodies (arrows)

**Figure 3:**
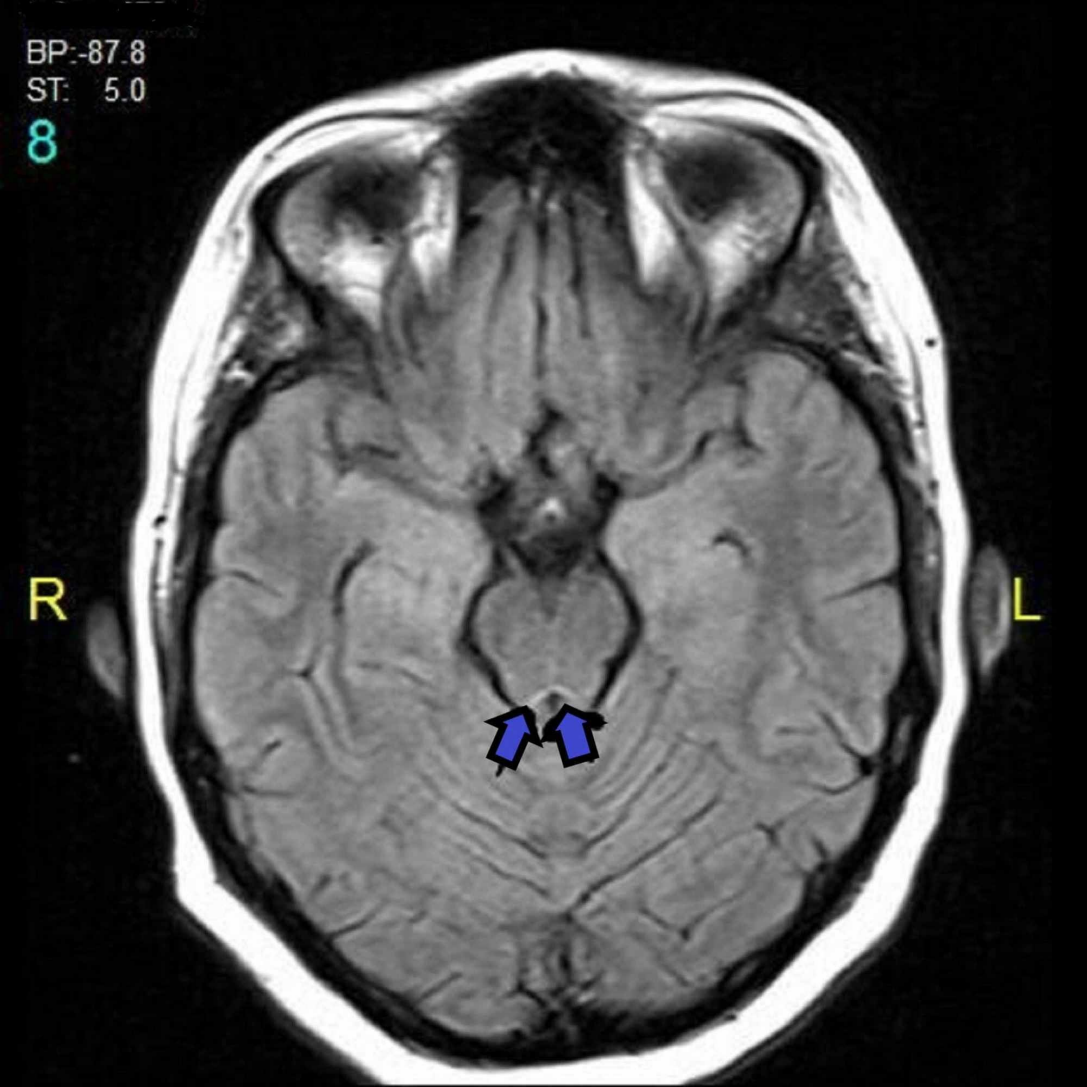
Hyper-intense signals in the peri-aqueductal area (arrows)

On laboratory investigations, she had hypokalaemia of 2.8 mmol/L and hypomagnesia of 0.68 mmol/L. Complete blood count and other electrolytes, including liver and renal function, were within normal limits. Her thyroid-stimulating hormone (TSH) was suppressed (0.01 milli-international units per liter), and she had increased free FT4 (28.3 μg/dL) and FT3 levels (6.9 μg/dL). CT scan of the head was not done because of her pregnancy. MRI of the head showed hyper-intense signals in periaqueductal grey matter, mammillary bodies, tectal plate around the third ventricles, and cerebellar hemisphere (Figures 1, 2, 3). These findings were consistent with WE.

The patient received thiamine 500 mg intravenously three times a day for three days, followed by 250 mg orally for 14 days. She had a complete clinical recovery and was discharged home safely. She went on to have an unremarkable pregnancy and a normal vaginal delivery with an unremarkable post-natal course.

## Discussion

WE is an acute neuropsychiatric condition caused by severe thiamine deficiency. Thiamine is an essential cofactor in many biochemical pathways in the brain. The overall incidence rate of WE is 0.04-0.13% [7]. HG is an infrequent cause of WE. WE occurring due to HG and hyperthyroidism is rare and challenging to recognize. This diagnostic dilemma arises from a relatively non-specific clinical presentation and the non-availability of a specific routine laboratory test.

WE is diagnosed in patients based on the presence of two of the following four criteria: 1. dietary deficiency; 2. oculomotor abnormalities; 3. cerebellar dysfunction; and 4. either altered mental status or mild memory impairment.

The diagnosis is mainly clinical and confirmed by characteristic MRI findings and dramatic clinical response to thiamine. A high index of suspicion is required for early diagnosis of WE in HG, and early initiation of thiamine supplementation is essential [8-10]. Bonucchi et al. have reported a case where the hypermetabolic state from thyrotoxicosis in a patient with HG contributed to the development of WE. The patient received IV dextrose without thiamine supplementation, which also contributed to the development of WE [11].

Our patient had altered mental status with memory impairment. She had cerebellar dysfunction as manifested by gait ataxia and hyporeflexia. She was also found to have electrolyte imbalance with characteristic MRI findings. The diagnosis of WE was confirmed by characteristic MRI findings and an excellent response to thiamine. We recommend keeping the WE in the differential when evaluating a patient with HG presenting with an altered mental state or cerebellar signs. Also, we are of the view that high-dose thiamine should be administered in patients with HG and impairment of higher mental function, oculomotor palsy, or cerebellar signs while looking for an alternative diagnosis.

## Conclusions

WE is a potentially reversible condition if diagnosed and treated early. Thiamine supplementation is crucial for women with HG. We emphasize the importance of prompt thiamine supplementation in pregnant women with prolonged vomiting, especially before starting intravenous or parenteral nutrition. We also underline the necessity of promptly replacing thiamine when neurologic symptoms or signs develop in a patient with HG.
